# Induced fit for cytochrome P450 3A4 based on molecular dynamics

**DOI:** 10.5599/admet.729

**Published:** 2019-12-11

**Authors:** Israel Quiroga, Thomas Scior

**Affiliations:** 1 Faculty of Chemical Sciences, Benemérita Universidad Autónoma de Puebla, Puebla, Pue., Mexico

**Keywords:** Drug metabolism, cytochrome P450, Cyp3A4, conformational selection, substrate selectivity, docking, RMSD, RMSF

## Abstract

The present study aims at numerically describing to what extent substrate - enzyme complexes in solution may change over time as a natural process of conformational changes for a liganded enzyme in comparison to those movements which occur independently from substrate interaction, i.e. without a ligand. To this end, we selected structurally known pairs of liganded / unliganded CYP450 3A4 enzymes with different geometries hinting at induced fit events. We carried out molecular dynamics simulations (MD) comparing the trajectories in a “cross-over” protocol: (i) we added the ligand to the unliganded crystal form which should adopt geometries similar to the known geometry of the liganded crystal structure during MD, and – conversely – (ii) we removed the bound ligand form the known liganded complex to test if a geometry similar to the known unliganded (apo-) form can be adopted during MD. To compare continues changes we measured root means square deviations and frequencies. Results for case (i) hint at larger conformational changes required for accepting the substrate during its approach to final position – in contrast to case (ii) when mobility is fairly reduced by ligand binding (strain energy). In conclusion, a larger conformational sampling prior to ligand binding and the freezing-in (rigidity) of conformations for bound ligands can be interpreted as two conditions linked to induced-fit.

## Introduction

Two major tenets have been established in the field of ADMET research worldwide. On the one hand, experimental work ever since has laid the groundwork and much of the extant literature evidence in the field has been gathered by either clinical studies or *in vivo* animal tests. On the other hand, during the last three decades efforts have been undertaken to reduce the need for animal tests, applying *ex vivo* or *in vitro* tests (isolated enzymes, cell-based, or living tissues, etc.) in tandem with *in silico* applications of biophysical techniques in pharmacokinetics and drug toxicology. Nowadays both tenets coexist in a complementary way all of which is exemplified by two recent drug inhibition studies of CYP targets: (i) animal tests with repaglinide and atazanavir [1], and (ii) computed inhibition models with organosulfur compounds [2].

Cytochrome P450 (CYP450 or CYP for short) belongs to a superfamily of monooxygenase enzymes that participate in the pathway of elimination of xenobiotics, including drugs in humans [3]. Certain CYP enzymes are capable of metabolizing a large variety of substrates of different sizes. They belong to a group of proteins with structural flexibility in addition to a huge cavity at the active site [4-8]. These enzymes are capable of adopting different conformations, both in their main chain and in their side chains, which allows the metabolic activity to be carried out on their various substrates; a phenomenon that in enzymes is known as induced fit. Prior to our present work, we laid the knowledge bases for induced fit and molecular flexibility of CYP and drugs in a survey article [9]. Induced fit describes the structural changes upon ligand - receptor binding. The ligand induces these changes at the binding site which normally constitutes a cavity in form of a deep cleft or a shallow surface depression. In our study the CYP enzyme binds its substrate for bio-transformation, here a drug. Induced fit also means that the geometry of the entrance to the binding site has a direct impact on the local shape (so-called active conformations) of the substrate. This matters in drug design when only three-dimensional models from free ligands (unbound drugs) and receptors (without ligands) have been reported, but the exact atom positions of the ligand - receptor complex are necessary to understand the molecular mechanism of action to design new drugs.

To simulate the effect that the induced fit has on the CYP activities in computational studies is a challenge. Albeit, some biophysical techniques have been proposed that would overcome the difficulties to simulate induced fit: (i) "*soft-docking*", is the deliberate reduction of repulsive forces; (ii) the management of side chain rotations using rotamer libraries; (iii) multiple structure modeling that considers the use of several crystal structures with different conformations of the same protein or related isoforms, or (iv) molecular dynamics (MD) calculations and the subsequent use of docking in a frame of the MD simulation that shows a favorable conformation [10-12]. MD simulates the structural flexibility of proteins as well as the characteristics that kept them stable.

**Figure 1.**
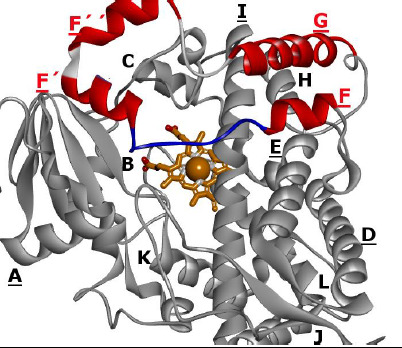
Display of human enzyme CYP3A4. The three-dimensional model is centered on the active site with the heme group with central atom Fe (brown). The segments are labeled by letters from A to L following the sequence from N- to C-term. The red color marks the F-G loop with its F’-F’’ segment. It was reported to undergo conformational changes upon ligand binding [14].

However, these techniques are not fool-proof and do not guarantee success. In some cases, neither crystal structures are at hand nor do rotamer libraries provide solutions to model the experimental data. In the context of our study hydroxylation patterns, CYP enzyme - substrate binding specificities, flexible protein segments or thermodynamic properties are amenable to computer-aided simulations [13-17]. Skopalik *et al.* compared the flexibility patterns of CYP3A4, CYP2C9 and CYP2A6 [14]. The researchers identified flexible loops adjacent to the active sites, e.g. B-C loop in CYP2C9 or F-G loop in CYP3A4 (Figure 1). Moreover, Chang *et al.* found that salt bridges, aliphatic and aromatic interactions between secondary structures of CYPs maintain their initial conformations [18].

In 2009 Seifert and Pleiss published a study on substrate specificities of CYP [5]. They reasoned that the sites of metabolism (SoM), i.e. which ligand atoms could be hydroxylated, correspond tightly to the conformational changes at the active sites. As a most valuable asset, predicting products and SoM for drugs by CYP enzymes was found amenable to *in-silico* approaches [15]. In this context we also explored the SoM of certain CYP substrates applying ligand docking and molecular dynamics techniques [19,20].

Crystallography does not always provide evidence for main or side chain movements since structure elucidation is carried out under solid phase package forces at very low temperatures and under destructive x-ray exposure of the crystal probes. Other model limitations are known as follows: (i) substrate size and adaptive volume of its cavity; (ii) resolution of the crystallographic image; (iii) crystallographic structure optimization and modeling to predict geometries for unresolved (highly flexible) areas; or (iv) 3D templates for homology modeling of unknown target structures [21-23].

And even point mutations further away from the active sites could alter the overall three-dimensional geometries – and subsequently affect the way of how CYP enzymes interact with their substrates [24,25].

On the other hand, Lampe and colleagues of the group of Ortiz de Montellano studied the conformational changes of liganded and unliganded CYP119 combining NMR and MD techniques [26]. The group found that weak binders to the active site allow the complex to move more than when stronger binders occupied the cavity at the active site. The authors conclude that substrate recognition and binding could be more a matter of conformational selection than a defined induced fit which could be described as the induction of a distinct conformational change in the receptor in the presence of a ligand. In the case of substrate - CYP complexes the substrate occupies the cavity at the heme site in a favorable orientation for its subsequent biotransformation. Recently, we examined the details of the P450 catalytic cycle elsewhere [9].

It is noteworthy to underscore that the aforementioned reports [4-8,10-17] do not undertake numeric measurements to reproduce the displacement of initial atom positions during MD production runs.

Root means square deviations of atom positions in space (RMSD for short) can be used to numerically reflect atom movements during a period of time in an MD simulation when following selected atoms. A larger RMSD value (typically 4 to 6 or 7) indicates that protein structures have moved more freely and have suffered larger geometrical shifts [27]. RMSD comparison for structural deviations against a template, however, is not fool-proof and other distance measurements have been proposed [28]. In particular, global RMSD measurements may reflect changes in irrelevant parts and not just at the center of interest; for instance, the movements may occur more around the active site.

To circumvent such drawbacks, any movement was under scrutiny, such that not only the heme site mattered but also all other protein parts did because any geometrical changes in liganded and unliganded proteins could hint at induced fit phenomena. We averaged the positions of each atom along the MD trajectory as a reference to calculate RMSD. This way, we obtained a trustworthy framework of the average positions of each atom of the protein because any larger magnitude of movement would be represented by higher RMSD values (Table 5 in the Results section). Besides RMSD, so-called heat maps of root mean square fluctuation were also analyzed.

Given the reported conundrum about geometrical changes in general and in the context of induced fit for CYP enzymes [9,21,26-28], we decided to study pairs of unliganded and liganded crystal complexes of the same CYP enzyme. This way, geometrical differences are known before MD simulation thanks to experimental determination (PDB data). Moreover, after relaxation to thermal equilibrium any changes in the observed geometries during MD simulation could be related to induced fit [29-33]. The silently made assumption here was that the structural differences between the liganded and unliganded crystal structures could no longer be due to crystal package forces after force field treatment to relax both protein structures (cf. aforementioned model limitations).

According to our literature survey [9], no publication considered the pairwise study of CYP enzymes in presence and absence of ligands and how this could affect their geometries. Based on what is known to date and not being explicitly documented in the extant literature, the need arises to systematically study the ligand effect on initial atom positions by computational MD and evaluate the numerical results in the context of induced fit.

## Experimental

### Structural input data

Molecular dynamics techniques were used to evaluate the conformational changes in the structure of the protein backbone. The MD results were evaluated based on the atoms’ start positions which were taken from the crystal structures.

### Selection of input structures

After inspecting pairs of crystal structures of human CYPs with and without drug substrates in their respective cavities in the protein database (PDB) [34], it was decided to use two PDB entries for human CYP3A4: 1TQN and 3NXU [29,30]. The former presents a single chain biological unit without substrate. In turn, 3NXU presents a homodimer with the antiviral drug ritonavir (RIT) as its substrate. RIT occupies the active site. For this study, we removed chain B from 3NXU. In 1TQN four amino acids (K282, E283, T284 and E285) are missing in a loop (H-I) not belonging to the active site while 3NXU shows unresolved residues in positions similar to that of 1TQN.

**Figure 2.**
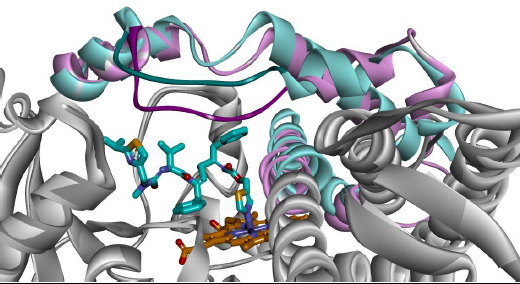
Superposition of a pair of liganded and unliganded enzyme pair. The two crystal structures of human CYP3A4 are displayed by backbone lines indicating helical and loop parts in gray color. On top two colors mark conformational differences in the F-G loops of unliganded 1TQN (magenta) and liganded 3NXU (bluish). The ligand is ritonavir (atom sticks in the center, blue C, dark blue N, red O). The catalytic heme group (brown sticks with O atoms in red and FE in violet/purple). All H atoms omitted.

This pair was chosen based on the following five reasons: (i) both structures were chosen because of the geometrical changes which can readily be ascribed to the presence or absence of ligand at the active site: in 1TQN the F-G loop is displaced inwards into the catalytic cavity, while in 3NXU the backbone and side chain geometries of this loop are relocated due to the presence of ritonavir (Figure 2); (ii) they are crystallographic works of different research teams; (iii) they have a similar resolution of high quality; (iv) 3NXU uses molecular replacement as a method to determine its structure, i.e. 1TQN was taken as a crystallographic reconstruction template; (v) atoms are not missing at their active sites and displacement of their main and side chains is clearly observable.

We measured the binding site volume to confirm the generally accepted view that hCYP3A4 can bind many drugs of different shapes and sizes thanks to its huge and variable cavity between 1440 and 2130 [Å^3^], cf. human CYP2C9, for example, has a smaller volume: up to 1400 [Å^3^] (PDB code: 1R9O) [20].

### Four combinations of enzyme and substrate structures as input for Molecular Dynamics

The following section describes how four input models for MD were created from a pair of PDB entries.

The two crystal structures were taken as start geometries for two input model without changes: (i) the chain A of the originally unliganded 1TQN [29] and (ii) the originally liganded 3NXU [30]. In addition, two more input models were generated to complete the cross-over design to study all four possible combinations systematically: (iii) an unliganded model which was generated from the originally liganded 3NXU by removing the ligand and leaving all spatial coordinates of the protein unchanged. Furthermore, another liganded 1TQN model (iv) was created from the originally unliganded 1TQN. Here the ligand’s start position was placed into the cavity near its observed pose (from 3NXU) near the heme group in a way which again left the Cartesian coordinates of the enzyme unchanged (Table 1).

### Descriptors and chemometrics for induced fit evaluation

MD simulations studied whether the geometries of the liganded or unliganded complexes will change during simulation time. Whether or not the model conserves or changes its overall structure can be measured as RMSD comparing the crystals geometries to the MD production geometries for the liganded and unliganded complexes. The RMSD is defined as:


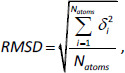


where *δ*_i_ is the distance between the atom i and the reference atom.

Using existing computational tools, it is possible to measure descriptors of the active site that allow us to correlate the induced fit effects with its biological activities. The VMD software was used to calculate the measurements between the model complexes [35].

### Parameterization and molecular dynamics

NAMD [36] software was used with CHARMM27 force field [37] and displayed by VMD [35] to carry out the MD runs and the equilibration described above.

The four input models described in Table 1 were solvated in a cubic water box with the water model TIP3P (1nm). The periodic boundary conditions were applied in all directions. The parameters for heme central atom (Fe) were set to coordinate oxygen moieties at 2.5 Å [38-40], and thereby maintaining a neutral charge between Fe and O with q= +2 and -2, respectively. Of note heme group atoms and reactive oxygen as a distal coordinated moiety were fixed in their experimentally observed positions as a positional reference to analyze MD runs. There were 100000 steps of energy minimization for all atomic coordinates. To balance each model the temperature was increased from 0 K to 310 K gradually for 0.25 ns. In the next stage, the balanced systems were subjected to 60 ns of production time at a time step of 2 fs at room temperature (310 K) and normal pressure (1 atm).

### Analysis of the results

Once the MD simulations were performed, it was expected that the MD geometries of the liganded and unliganded input models will merge into similar geometries of the crystal structures 3NXU or 1TQN. In turn, it was also expected that both liganded models undergo restricted movements of their side chains, seen as smaller RMSD values. The two models without substrate would be similar to crystal structure 1TQN. But with smaller RMSD values for the unliganded model based on the originally unliganded 1TQN, and conversely the need of larger rearrangements in space (more mobility) with fairly higher RMSD values of the unliganded model stemming from the originally liganded 3NXU (Table 2).

So far, our analysis does not account for trajectory differences. To tackle this issue, too, we compared RMSD in a pair-wise manner between the four MD models to detect some portion of induced fit. After all, the two molecular systems with ritonavir had to show higher similarities compared the two models without it.

The cross-over study design and the pairwise RMSD comparison helped assessing the mobility or rigidity effect that the substrate had on human CYP3A4. As a most valuable asset, the approach allows not only evaluating the effect of initial positions of the crystal atoms in the MD studies but also testing the extent of conformational changes with the subsequent interpretation as induced fit for the enzymatic activities of CYP targets.

## Results and Discussion

The spatial difference between a pair of corresponding crystal structures (PDB codes: 1TQN and 3NXU) was measured in a first step. Effectively, the pair constitutes one of the rare cases where the same CYP enzyme is available in liganded and unliganded states such that their geometrical difference can be interpreted as induced fit after reducing crystal package strain energies by relaxation to equilibrium state under the CHARMM force field (cf. details about assumptions or limitations). Of note, two RMSD types were calculated: (i) with only main chain atoms, or (ii) with all atoms except hydrogen atoms. Both types of calculations were carried out on either the entire protein or at the active site. The latter comprises the literature-known segments from residue H54 to F60, S100 to E122, T207 to P218, I238 to E244, V296 to S311 and F367 to R375. Then, all possible pairs of RMSD were compared and special attention drawn to the overall mobility and the F-G loop displacements for substrate recognition (Table 3). The geometrical changes at the active sites of 1TQN and 3NXU were documented, too (Table3). Both represent the same enzyme, but their major structural differences could be pinpointed to only those residues which interacted with ritonavir.

Following Table 2, we expected the unliganded (liganded) models to merge into geometries similar to 1TQN (3NXU) during MD simulations. Intriguingly, the MD results in Table 4 fell short of expectation. The absence of RIT allows CYP3A4 to explore the conformational space much more than the liganded enzyme which resembles more the crystal structure 3NXU (Table 4).

**Figure 3.**
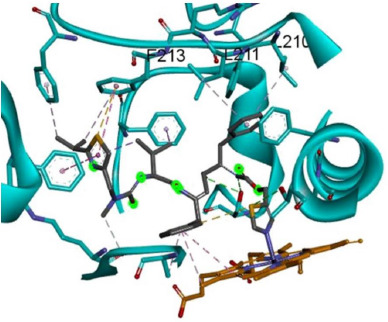
Intermolecular interactions between ritonavir substrate and CYP3A4 enzyme (3NXU). The ligand’s favourable intermolecular interactions at the active site stabilized the complexes (1TQN+RIT and 3NXU+RIT). The noncovalent bonds (intermittent lines) between ligand and heme or amino acids decrease significantly the protein flexibility.

Playback analysis by VMD showed that the liganded models follow trajectories in a much shorter range of near-site atomic displacements, i.e. their average geometries lie closer to the starting geometries. In more general terms, ligand - receptor complexes are less flexible by nature, due to ligand-mediated non-covalent bonds at the active site (Figure 3).

**Figure 4.**
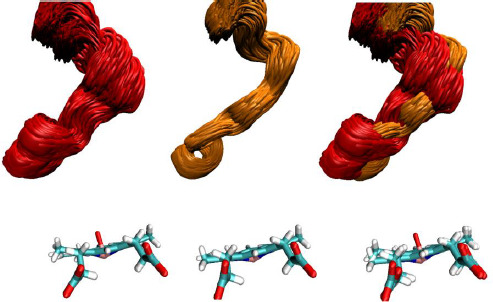
Superposition of MD conformations with the F-G loop near the active site. Model 3NXU-RIT (left) and liganded 3NXU (center). Both models were aligned in space (right). Liganded 3NXU (center) had a much smaller mobility range than 3NXU-RIT (left) during dynamics simulation.

The presence of RIT at the active site had already induced a larger geometrical shift (Figure 4 and Table 5). This conclusion is in total keeping with the experimentally observed huge RMSD differences between ligand-bound and unbounded crystal structures: RMSD 3.8 at the active site or RMSD 1.6 for the entire complex. The two moving F-G loops from 3NXU with or without ligand were superimposed to display the difference in mobility throughout the entire dynamic simulation (Figure 4). Again, with RIT in the binding cleft smaller displacements were noticed (Figure 3). The presence of RIT exercises a twofold effect: (i) preventing the F-G loop from occupying positions close to the heme group, and (ii) intermolecular interactions stiffening the loop architecture.

**Figure 5.**
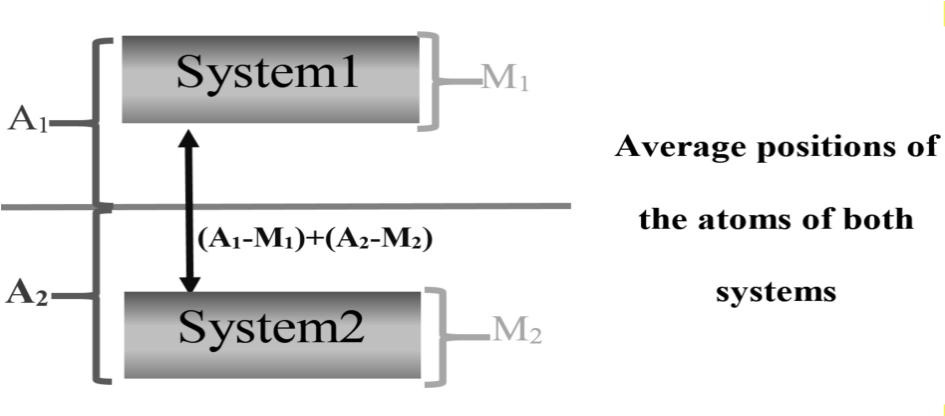
Averaged RMSD calculation with the equation (A_1_-M_1_) + (A_2_-M_2_). M is the RMSD that expresses the movement of a system (**Table 5**). A is the RMSD of a system when compared to the average positions of the atoms of both systems. The higher the calculated value, the more structural differences two systems have in an MD run.

In Table 5 the differential behavior of RIT-bound *versus* unbound systems becomes evident with less flexible main chains for the former and high flexibilities for the latter. It is, however, possible to measure the overall geometric similarities without the dynamic contributions after assessing corresponding differences between the four molecular systems in Table 1. To get rid of RMSD bias due to movement restrains of more stable liganded complexes, the RMSD of any two models (now called System1 or System2, respectively) was calculated taking as reference the average value of corresponding atom positions from both systems. The calculated RMSD of System1 with respect to the average positions of both systems taken together is called A_1_, and the calculated RMSD of System2 respect to the average positions of both systems is called A_2_. RMSD is amenable to recalculation thanks to the fact that all systems have the same total number of atoms in their data files. Therefore, even ever higher A values (Figure 5) were met in a system, the greater the dynamic and structural differences would be. The equation (A_1_-M_1_)+(A_2_-M_2_), where M represents the RMSD for the unbiased movement of a system (Table 5), allows recalculating the structural similarities between two systems in a way which is independent of their intrinsic (own) movements (Figure 5 ). This way, it was possible to evaluate similarities for all model pairs: (i) the higher the values, the less structural similarities during simulations; (ii) conversely, the lower the values, the higher the outcome of similar conformations during MD.

The results of the recalculated RMSD were also summarized (Table 6). A higher RMSD in columns 5 and 6 of Table 6 expressed which of the two systems moved more relative to the other one. If both systems moved in a similar way they would have comparable RMSD values. As indicator of changes only atoms belonging to the active site and the main chain atoms were considered.

Upon calculating the difference between A_1_ and A_2_ (Figure 5), it becomes evident that the lowest dynamic differences belong to the pairs 1TQN+RIT and liganded 3NXU, and unliganded 1TQN and 3NXU-RIT. Another bias (unfair treatment of numeric data) was detected, namely the initial atom position dependence for the developing trajectories as shown by the values in column 7 in Table 6. Those MD data were the ones that had the greatest structural similarities when generated from the same input model. Precisely, this finding paved the way to work-around the initial position bias by recalculating RMSD of System1 and System2 (the liganded and unliganded models).

**Figure 6.**
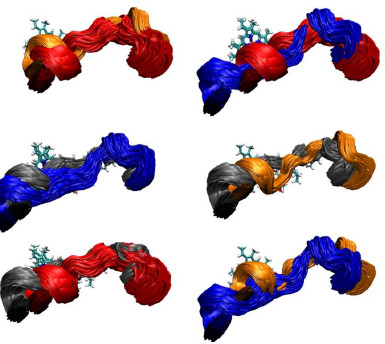
Superposition of MD conformations for the the F-G loop. The F-G loops of our four models are compared. They lies in close proximity to the heme group at the active site. First row to the left: the 3NXU-RIT systems (red) superimposed with 3NXU+RIT (orange). Second row to the left: 1TQN-RIT (blue) superimposed with 1TQN+RIT (black). Bottom row to the left: 1TQN+RIT (black) superimposed with 3NXU-RIT (red). First row to the right: 3NXU-RIT (red) superimposed with unliganded 1TQN (blue). Second row to the right: 1TQN+RIT (black) superimposed with 3NXU+RIT (orange). Bottom row to the right: 1TQN-RIT (blue) superimposed with 3NXU+RIT (orange).

Figure 6 shows illustrations of the model dynamics and representative (averaged) structures thereof. In particular, it displays the superpositions with the F-G loop. Systems with ritonavir have a smaller range of movement than those without ritonavir in the superposed region and elsewhere occupy different locations (midsection in Figure 6).

**Figure 7.**
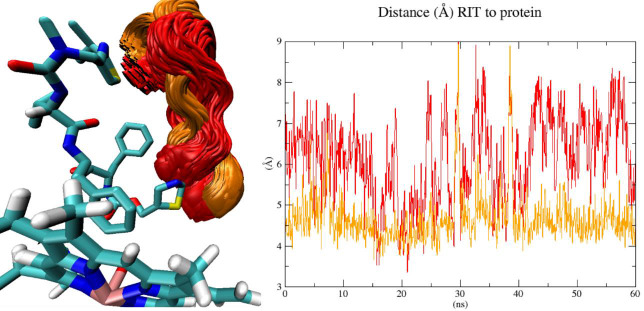
**Left panel**: Superposition of MD conformations (left) with the F-G loop near the active site of 3NXU-RIT (red) or liganded 3NXU (orange). The liganded 3NXU (orange) lies in closer proximity to heme than ritonavir during MD. **Right panel**: Root mean square distance over time plot (RMSD) for the distance between ritonavir and F-G loop during MD. Of note, lacking substrate the coordinates, the unliganded models (here: 3NXU-RIT) the corresponding atoms of the crystal structure were used as reference to calculate RMSD. The minimum distances between references coordinates and protein of both systems (3NXU-RIT and liganded 3NXU) were assessed during 60 ns.

Upon superposition of 3NXU-RIT with 3NXU+RIT (right side of Figures 4 and 6), the occupied and unoccupied spaces at the active sites can be distinguished, as it is the particular case with the F-G loop of 3NXU+RIT (Figure 7, orange). It is located nearby the heme group and ligand RIT, while the F-G loop of the 3NXU-RIT system (Figure 7, red) is displaced to a distinct location above the F-G loop of 3NXU+RIT. The MD simulation allows us to infer that this F-G loop displacement of human CYP3A4 into an energetically more favorable conformation fairly reduces strain energy by steric hindrance. The underlying geometrical changes in side chains and backbone reflect the enzyme’s capability to metabolize substrates with an even larger volume than RIT – all of which leads to the well-known flexibility ascribed to CYP enzymes in general. As a direct result, our MD simulation provides computed evidence for induced-fit between receptor and ligand (here enzyme and substrate).

**Figure 8.**
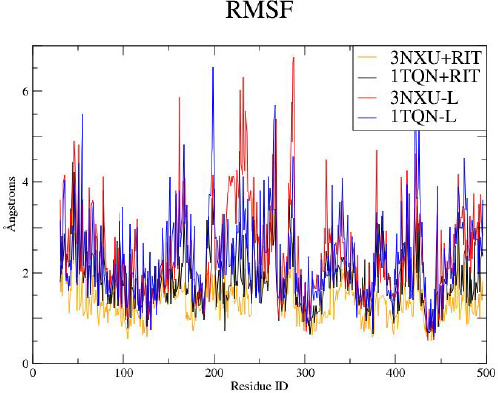
Root mean square fluctuation plot (RMSF). The X axis represents the residues of the primary sequence. The Y axis quantifies the corresponding movement of each residue measured in [Å]. The higher the Y value, the higher the mobility of adjacent residues in segment of the protein. The four combinations of the liganded / unliganded pair are color coded (inlay panel).

Comparing the heights in the plot of the root mean square fluctuation (RMSF) reflects changing flexibilities within the protein sequences (Figure 8). The lower fluctuations stem from 1TQN+RIT and 3NXU+RIT. More rigid regions can be spotted between them, too. The highest peaks are similar sequence regions, between residue ID 240, 270, 282, 95 and 44. In good keeping with the expected binding relevance of the F-G loop, a mayor geometrical shift had occurred when placing the drug into the unbound enzyme structure (1TQN+RIT). This constitutes a huge fluctuation near residue ID 200. The unbound start conformation had to adopt geometries (binding conformations) to accommodate the ligand. Intriguingly, unresolved segments in the crystal structure (here regions at residue ID 270 to 282) reflect low complexity areas and coincide with higher fluctuations all of which nicely explain why they were missing in the crystal structure.

In a more general view, MD studies simulate molecular movements in water solution under thermodynamic conditions which are relevant in biological systems, but other aspects may become important too: solvent entropies from bulk water and water-exclusion zones in lipophilic pockets or shallow surfaces, enthalpies from destabilizing hydrogen bonding, etc. In sight of such model limitations, trajectory interpretation was undertaken with utmost caution. Moreover, the physical and chemical micro-conditions (temperature and molecular neighborhood effects) at the moment of crystallogenesis or crystallography are fairly different from the physiological ones in living cells all of which can cause artifacts of (starting) geometries [21]. In our case, the average positions of the F-G loop of CYP3A4 of the unliganded 1TQN system could not approach the central heme group, giving way for a larger cavity and yielding higher geometric similarities with the crystal structure of CYP3A4 (3NXU-RIT). This is in keeping with published findings [26] because our RMSD data showed that the movements of enzyme-substrate complexes become more restricted than their unliganded counterparts.

## Conclusions

The induced fit or conformational selection process between drug and cytochrome P450 enzymes (CYP) was studied by molecular dynamics simulations. The study demonstrates that the smallest dynamic differences are met in pairs which share either the presence or the absence of bound substrates. Indeed, our pairwise study design unraveled that substrate alone does provide an effect on the geometric changes of the enzyme and that they can be observed and measured in molecular dynamics studies. The computed trajectories were parameterized as RMSD reflecting spatial rearrangements of the start geometries which were taken from crystal structures of substrate-bound and unbound states of identical proteins. To this end, crystal structures of known liganded/unliganded pairs had been gathered prior to simulation. Precisely, comparing averaged RMSD data (originally liganded 3NXU: 0.9 < liganded 1TQN < unliganded 3NXU: 1.55 < originally unliganded 1TQN: 1.9) lent insight into hidden driven forces leading to geometrical changes for substrate recognition of human CYP3A4 proteins. For a worst-case scenario, we would state that both unliganded models did not follow a trajectory with F-G loop rearrangements to merge into the observed structure of the originally unliganded protein (1TQN). This finding, however, attests to the well-known conformational flexibility of the CYP family in general, reflecting the need for flexibility for successful ligand binding. Hence, the not merging trajectories into crystal geometries simulate a natural (intrinsic) behavior here. Conversely, ligand binding fairly reduced the flexibility of the complex. In a natural situation the active site has evolved to strengthen the substrate attachment until its biotransformation took place (oxidation, hydroxylation). Interpreting in this way our results, the simulations come close – if not – reflect properly natural behavior, all of which is our best-case scenario where MD is capable of reproducing induced fit of CYPs.

All told, it seems not far-fetched to assume that averaged RMSD captures the essence of conformational changes of induced fit. MD estimates the necessary changes for substrate recognition and regioselectivity. The results on theoretical grounds further the awareness for induced fit mechanisms upon ligand binding. Our work contributes to describe induced fit which has been frequently cited in the extant literature body but poorly described at an atomic scale.

## Figures and Tables

**Table 1.** Three-dimensional molecular models created to study induced fit for human CYP3A4. The label “+RIT” symbolizes the presence of ritonavir at the active site, regardless whether the position of RIT was taken from crystal positions or computed. The “-RIT” (minus ligand) was used in case of unliganded enzymes, i.e. the ligand is absence.

	Complexed enzyme	Uncomplexed enzyme
**Observed (X-ray) systems**	PDB Code: 3NXU, liganded receptor, the ligand is ritonavir: (i) 3NXU+RIT	PDB Code: 1TQN, unliganded receptor: (ii) 1TQN-RIT
**Modeled systems**	(iii) 1TQN+RIT	(iv) 3NXU-RIT

**Table 2.** The four MD simulations and their expected outcome in terms of end geometries. The starting positions are related with **Table 1.** Comparison of the protein structures of the four systems after the MD run with the original protein structures of the crystals reported in PDB 1TQN and 3NXU.

Type of 3D-model	Start geometry (System)	Enzyme geometric similarity with PDB	Interpretation
**Liganded Enzyme**	(iii) 1TQN+RIT	3NXU	Induced fit
**Liganded Enzyme**	(iii) 1TQN+RIT	1TQN	No induced fit
**Liganded Enzyme**	(i) 3NXU+RIT	1TQN	No induced fit
**Liganded Enzyme**	(i) 3NXU+RIT	3NXU	Induced fit
**Unliganded Enzyme**	(iv) 3NXU-RIT	1TQN	Induced fit
**Unliganded Enzyme**	(iv) 3NXU-RIT	3NXU	No induced fit
**Unliganded Enzyme**	(ii) 1TQN-RIT	3NXU	No induced fit
**Unliganded Enzyme**	(ii) 1TQN-RIT	1TQN	Induced fit

**Table 3.** The computed value of the root-mean square distance (RMSD, in Å) between the original crystal structures of PDB 1TQN and 3NXU comparing the entire protein and only the region corresponding to the active site. The calculation of both regions was taking into account the atoms corresponding to the main chain and later to the atoms that were not hydrogens.

Compared region	RMSD value (Å)
The whole protein	1.6
Active site	3.8

**Table 4.** Computed RMSD values [Å] for 1TQN and 3NXU. The averaged values represent a movement corresponding to an area around the active site compared against all positions during the entire simulation. The main chain and all non-H atoms in the area were compared.

Compared region:	whole protein		active site	
Reference PDB crystal	**1TQN**	**3NXU**	**1TQN**	**3NXU**
RMSD of system **(iii) 1TQN+RIT**	**1.5**	2.1	**1.5**	3.8
RMSD of system **(ii) 1TQN-RIT** (orig.)	2.2	2.8	3.9	5.1
RMSD of system **(i) 3NXU+RIT** (orig.)	2.2	**1.2**	4.0	**1.1**
RMSD of system **(iv) 3NXU-RIT**	2.2	1.7	3.8	2.4

**Table 5.** Calculated RMSD values [Å] for unliganded 1TQN, 1TQN+RIT, 3NXU-RIT and liganded 3NXU. The averaged values represent a movement corresponding to an area around the active site compared against all positions during the entire simulation. The columns SD, min and max show the values of standard deviation SD, RMSD minima and maxima.

hCyp3A4 model	RMSD [Å]	SD	Min [Å]	Max [Å]
**1TQN+RIT**	**1.2**	0.36	0.32	3.59
**1TQN-RIT** (orig.)	2.0	0.66	0.36	5.07
**3NXU+RIT** (orig.)	**1.2**	0.38	0.20	2.74
**3NXU-RIT**	2.2	0.84	0.29	5.35

**Table 6.** Structural and dynamic differences in the modeled systems for this study (1TQN-RIT, 1TQN + RIT, 3NXU-RIT and 3NXU + RIT) measured as RMSD (in Å). The comparison between the four systems is made among themselves taking into account the six possible combinations of **Table 2.** M_1_ and M_2_ are the values of RMSD for the calculated movement of systems 1 and 2 respectively in **Table 5.** A_1_ and A_2_ represent the RMSD of systems 1 and 2 respectively, having as reference the average positions of the atoms of both systems. (M_1_-A_1_) + (M_2_-A_2_) measures the differences between systems 1 and 2.

System1	System2	M_1_	M_2_	A_1_	A_2_	(A_1_-M_1_)+(A_2_-M_2_)
3NXU-RIT	3NXU+RIT	2.2	1.2	1.5	1.2	-0.6
1TQN-RIT	1TQN+RIT	2.0	1.2	1.7	1.5	0.0
1TQN+RIT	3NXU-RIT	1.2	2.2	1.8	2.0	0.4
1TQN-RIT	3NXU-RIT	2.0	2.2	2.5	2.5	0.8
1TQN+RIT	3NXU+RIT	1.2	1.2	2.0	2.0	1.6
1TQN-RIT	3NXU+RIT	2.0	1.2	2.7	2.6	2.2
